# The PpPep2-Triggered PTI-like Response in Peach Trees Is Mediated by miRNAs

**DOI:** 10.3390/ijms252313099

**Published:** 2024-12-05

**Authors:** Laura Foix, Maria Pla, Beatriz Martín-Mur, Anna Esteve-Codina, Anna Nadal

**Affiliations:** 1 BETA Technological Center (TECNIO Network), University of Vic-Central University of Catalonia (UVic-UCC), Carretera de Roda 70, 08500 Vic, Spain; laura.foix@udg.edu; 2 Institute for Agricultural and Food Technology, Universitat de Girona, C/ Maria Aurèlia Capmany, 61, 17003 Girona, Spain; maria.pla@udg.edu; 3 Centre Nacional d’Anàlisi Genòmica (CNAG), C/ Baldiri Reixac 4, 08028 Barcelona, Spain; beatriz.martin@cnag.eu (B.M.-M.); anna.esteve@cnag.eu (A.E.-C.); 4 Parc Científic de Barcelona, Universitat de Barcelona, C/ Baldiri Reixac, 4, 08028 Barcelona, Spain

**Keywords:** plant elicitor peptide (Pep), plant defense, *Prunus*, miRNA sequencing, differential network analysis, gene set enrichment analysis

## Abstract

Plant diseases diminish crop yields and put the world’s food supply at risk. Plant elicitor peptides (Peps) are innate danger signals inducing defense responses both naturally and after external application onto plants. Pep-triggered defense networks are compatible with pattern-triggered immunity (PTI). Nevertheless, in complex regulatory pathways, there is crosstalk among different signaling pathways, involving noncoding RNAs in the natural response to pathogen attack. Here, we used *Prunus persica*, PpPep2 and a miRNA-Seq approach to show for the first time that Peps regulate, in parallel with a set of protein-coding genes, a set of plant miRNAs (~15%). Some PpPep2-regulated miRNAs have been described to participate in the response to pathogens in various plant–pathogen systems. In addition, numerous predicted target mRNAs of PpPep2-regulated miRNAs are themselves regulated by PpPep2 in peach trees. As an example, peach miRNA156 and miRNA390 probably have a role in plant development regulation under stress conditions, while others, such as miRNA482 and miRNA395, would be involved in the regulation of resistance (R) genes and sulfate-mediated protection against oxygen free radicals, respectively. This adds to the established role of Peps in triggering plant defense systems by incorporating the miRNA regulatory network and to the possible use of Peps as sustainable phytosanitary products.

## 1. Introduction

Plants have a sophisticated defense system that is triggered by the perception of molecular patterns associated with pathogens (PAMPs) and endogenous danger signals (DAMPs) through transmembrane pattern recognition receptors (PRRs) [1,2].

Plant elicitor peptides (Peps) are approximately 23 to 40 amino acids long secondary DAMPs cleaved out of precursor proteins (PROPEPs) that are released to the apoplast following cell disruption due to pathogen attack or wounding. They are recognized by specific Leucine-Rich Repeat Receptor-Like Kinase (LRR-LRK) transmembrane Pep receptors (PEPRs), which result in the induction and amplification of pattern-triggered immunity (PTI, i.e., the first line of inducible plant defense). PTI involves common signaling molecules, e.g., Ca^2+^, ROS and hormones (primarily salicylic acid, SA, ethylene, ET and jasmonic acid, JA), and extensive transcriptional reprogramming [2,3,4,5,6]. The overexpression and exogenous application of Peps at nanomolar concentrations increases resistance to various bacterial or fungal pathogens and herbivore attack [7,8,9,10,11] in ex vivo experiments and in planta [7,9,10,12,13,14]. As an example, the preventive treatment of peach plants with 1 μM doses of the *Prunus persica* peptide PpPep2 resulted in about 50% reduction in the symptoms following massive infection [3] with the bacterial pathogen *Xanthomonas arboricola* pv. *pruni* (*Xap*) [7].

PROPEP and PEPR orthologue genes are widely present within the angiosperms [15,16]. Sequences from the same plant family cluster together and family-specific motifs have been identified. Thus, there is inter-family incompatibility in their activity. The signal transduction pathways, however, are analogous.

Transcriptome deep sequencing analyses in *Arabidopsis* and *P. persica* (using AtPep1, and PpPep1 and PpPep2, respectively) showed that the topical application of Peps mimics the PTI natural response [7,17]. This includes the quick induction of PEPRs, PRRs and intracellular receptors, regulatory genes such as those related to hormone signaling (ET, JA, ABA) and transcription factors, calcium signaling proteins, plant disease resistance R proteins, etc., and the slower induction of pathogenesis-related proteins and cell wall-related genes.

There is a clear parallelism in the transcriptome dynamics caused by exogenous application of Peps and pathogen attacks, mostly affecting the same types of genes, although the latter showed an overall slower response, possibly due to the infection progress [7]. Thus, PTI induction is postulated as the Pep mode of action to achieve plant protection.

Complementary to protein coding genes, noncoding RNAs (ncRNAs) play essential regulatory roles in plants, particularly in development and in the response to abiotic and biotic stresses [18,19,20,21]. Micro RNAs (miRNAs) are 20–24 nucleotides small endogenous ncRNAs. They are transcribed from MIR genes into a stem-loop structured primary miRNA (pri-miRNA), which can be processed to shorter precursor pre-miRNAs and subsequently into miRNA/miRNA* duplexes. MIR transcription depends on a complex regulatory mechanism that involves numerous factors and microprocessor elements. miRNA/miRNA* duplexes are loaded on ARGONAUTE1 (AGO1) to constitute the RNA-induced silencing complex (miRISC), which is translocated to the cytosol. After that, the miRNA* is removed from miRISC, leaving the functional miRNA to target complementary mRNAs for degradation and/or translational silencing, thus contributing to gene regulation [22,23,24,25,26,27,28,29,30]. miRNAs can be functional within the same cell and can also be exported to distant cells.

miRNAs have been involved in plant immunity to bacteria and other pathogens [20,29,31,32,33,34,35] by regulating PTI and/or effector-triggered immunity (ETI). This includes pathogen perception through plant receptors, signal transduction and downstream immune responses, e.g., reactive oxygen species (ROS) accumulation, transcription factors and pathogenesis-related (PR) gene expression, callose deposition and plant hormone regulation. Some miRNAs are known to silence the auxin signaling pathway, which results in the activation of PTI [36,37] while some others contribute to fine-tuning the defense response [19].

The exogenous application of the PAMP flg22 onto *Arabidopsis* plants has an effect on the levels of various miRNAs, in coherence with miRNAs having a role in PAMP signaling [36,37]. Given the similarity of downstream events in response to PAMPs and DAMPs, we hypothesized that exogenous treatments with DAMPs might as well have an effect on the levels of certain miRNAs. We thus envisaged further characterization of the miRNA response to treatment with the DAMP PpPep2 onto *P. persica*, taken as an example. Peps are good candidates to establish natural, environmentally friendly and targeted culture management strategies. Improved knowledge on its mechanism of action is an important step towards transference of this technology. Here, we analyzed miRNA profiles of peach trees upon PpPep2 treatment using the application conditions that provide optimal protection against massive *Xap* infection.

## 2. Results and Discussion

### 2.1. miRNA-Seq Characterization of Peach Response to PpPep2

To assess the possible role of miRNAs in the response of peach to preventive treatment with PpPep2, we treated the leaves of juvenile plants with 1 μM of this peach peptide and carried out miRNA-Seq after 1 and 24 h using untreated plants as control. We previously showed that this is the optimal dose to protect peach trees against *Xap* [10] and that peach leaf transcriptome is strongly modulated 1 and 24 h after PpPep2 application [7]. One hour after PpPep2 application, there was a strong transcriptional regulation affecting up to 5% peach genes (i.e., differentially expressed genes, DEGs), with more than 90% upregulated. One day after PpPep2 treatment, most of these genes were downregulated to recover their levels before treatment, and there was ca. 1% novel DEGs. Transcriptional differences between the 24 h and the 48 h time points were less significant.

Nine miRNA libraries were constructed that corresponded to three experimental replicates of each, the control and 1 and 24 h time points, each consisting of nine plants and five leaves per plant. Data supporting this analysis are available in the Gene Expression Omnibus (GEO) repository, record GSE214135 (https://www.ncbi.nlm.nih.gov/geo/query/acc.cgi?acc=GSE214135; accessed on 1 December 2024).

Quality control and mapping statistics of the miRNA analysis are summarized in Table S1. We obtained 117, 267 and 647 raw reads that corresponded to an average of 12, 11 and 16 million reads 1 and 24 h after PpPep2 application and control, respectively. Moreover, 92% of pre_miRNA genes annotated in the *P. persica* NCBIv2.38 genome produced any read pair in any analyzed sample, while 16 of 210 annotated genes (i.e., 8%) did not produce any read pair. A total of 157 of the annotated pre_miRNAs (i.e., 75%) were consistently detected in all three replicates of at least one condition and reached values up to ~10^5^ normalized counts (Table S2).

**Figure 1:**
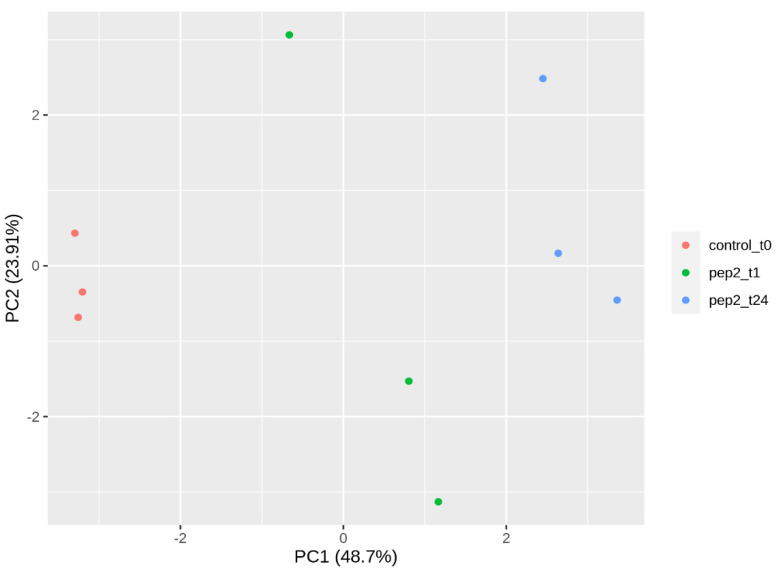
Principal component analysis (PCA) of log2 normalized expression data from the top 50 most variable miRNAs (DESeq2 1.20.0 package, [38]). Two principal components, PC1 and PC2, are represented. They have Eigenvalues above 1 and explain 48.7% and 23.91% of the variability, respectively. Colors represent the different peptide treatments: orange, time zero control (t0); green, 1 h PpPep2 (t1); and blue, 24 h PpPep2 (t24). Three biological replicates per sample are shown.

The processed data’s principal component analysis (PCA) (Figure 1) reveals that the main miRNA transcriptomic changes can be attributed to the Pep2 treatment and time-course condition. Untreated and the two Pep2-treated samples are widely separated across the main component (PC1), which explains up to 48.7% of data variability. It might be speculated that the variability within 1 h of biological replicates observed in PC2 (Figure 1) is linked to quick changes in miRNA levels occurring around this time.

**Figure 2:**
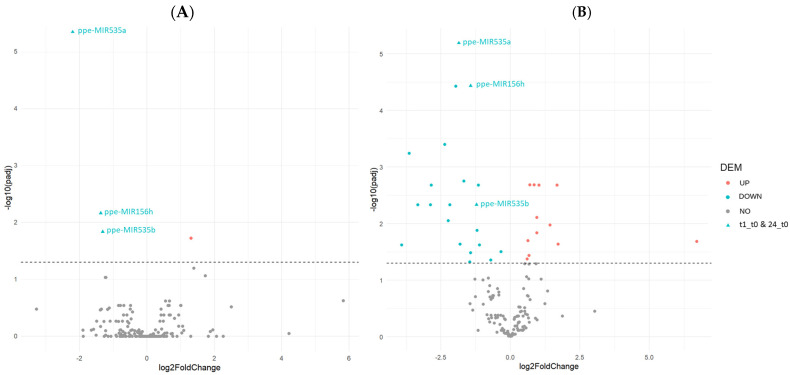
Volcano plots showing miRNA expression (expressed as log2FoldChange) 1 h after PpPep2 application in comparison to untreated control (**A**) and 24 h after PpPep2 application in comparison to untreated control (**B**); and the adj. *p*-value of the same comparisons (expressed as log10[adj. *p*]). The dashed line delimits differentially expressed miRNAs (DEMs), shown in red (upregulated DEMs, log2FC > 1) and blue (downregulated DEMs, log2FC < 1), from unregulated miRNAs (gray) using adj. *p* < 0.05. DEMs regulated at both comparisons are named and represented in triangles.

Differential expression analysis was performed to compare samples obtained 1 h or 24 h after peptide treatment with control samples. There were 33 differentially expressed miRNAs overall (DEMs, adj. *p* < 0.05) including the ones found in the comparisons of both time points against the control group (Figure 2, Table S2), which represents up to 15% of annotated *P. persica* miRNAs in the Ensembl Plants database. Thus, miRNAs seem to be involved in the peach response to PpPep2, which is in coherence with their role in plant immunity.

Except for one DEM, all were regulated 24 h after peptide application, with only three being regulated as early as one hour after the treatment. The remaining DEM was transiently upregulated one hour after PpPep2 application (Figure 2, Table 1). In response to PpPep2, 40% of DEMs were upregulated and 60% were downregulated. This is in contrast with the quick and massive mRNA changes observed in peach leaves in response to the same PpPep2 treatment. One hour after PpPep2 application, there was a peak in regulation affecting 1255 peach genes (ca. 5% of the total *P. persica* genes), with the vast majority upregulated and regaining normal levels within the next 24 (in some cases 48) h. Regulation was first detected 24 and 48 h after peptide application for a much smaller number of DEGs (i.e., 1.2% and 0.8% peach genes, respectively) [7]. The same pattern was described in *Arabidopsis* after treatment with the corresponding AtPep2, regulating up to 17% of genes two hours upon peptide application and 2.5% of genes ten hours later [17].

The 33 DEMs belong to 18 families: ppe-MIR156, ppe-miR159, ppe-miR162, ppe-MIR166, ppe-MIR167, ppe-miR168, ppeMIR169, ppe-miR171, ppe-MIR172, ppe-MIR390, ppe-MIR395, ppe-miR396, ppe-miR403, ppe-MIR482, ppe-miR530, ppe-MIR535, ppe-miR7122 and ppe-miR8127 (Table 1 and Table S2). It is known that miRNA molecules derived from many of these DEMs take part in the response to diseases caused by bacteria (e.g., miR156, miR159, miR167, miR169, miR390, miR482), fungi (miR156, miR168, miR396, miR482), viruses (e.g., miR159, miR171, miR395, miR482) and parasitic nematodes (e.g., miR167a) (see a review in [39] and references therein). This is consistent with their involvement in the PpPep2-driven defense response. Despite the known role of PAMPs and DAMPs in eliciting PTI, as well as the role of miRNAs in fine-tuning the defense response, only Li and colleagues (2010) [19] identified miRNAs involved in PAMP-triggered plant innate immunity using flg22 and *Arabidopsis thaliana*. When flg22 was applied, a number of miRNAs were found to be regulated and to bind AGO1. Here, we show that some of these flg22-regulated miRNAs (e.g., miR156, miR167, miR168, miR169, miR396) were also regulated by the DAMP PpPep2 in peach. Thus, there is some correlation between the miRNA response to PAMPs and DAMPs. Nevertheless, we identified a number of DEMs specific to PpPep2, notably those belonging to the ppe-MIR395 family but also those belonging to the ppe-miR171, miR7122, ppe-miR530, ppe-MIR535 and ppe-MIR166 families.

### 2.2. Analysis of Peach miRNA Response to PpPep2 on the Level of Processes

Mature DEM sequences are what constitute functional miRNA molecules. The mature DEM sequences were deduced using a combined analysis of two different databases i.e., Ensembl, encompassing the peach genome, and miRBase, compiling *P. persica* miRNAs. Note that, for six DEMs, i.e., ppe-MIR7112a and -b, ppe-MIR482a and -c, ppeMIR8127 and ppe-MIR395a, there were two different possible mature sequences that corresponded to the forward (5p) and the complementary (3p) strands, respectively. In these cases, the two sequences were considered potential functional miRNAs for further analyses. In consequence, there were 39 deduced mature miRNAs regulated by PpPep2. The majority (74% of them) were 21 nt long, 22% had 22 nt and the remaining 5% were 20 nt long.

Then, psRNATarget [40] was used to conduct target prediction analysis based on these 39 sequences. Mature miRNAs direct the RISC to target genes through base pairing [41]; hence, target prediction is dependent on sequence homology and secondary structure. The psRNATarget algorithms also consider the fact that, in plants, a single target gene can be targeted by multiple miRNAs and that a single miRNA can have a large number of target genes [42]. Using a *p*-value threshold of 0.05, we identified a total of 2431 DEM predicted target genes (DEM-Ts). A range of 45 to 117 DEM-Ts were predicted for each DEM, with varying degrees of targeting probability based on miRNA and target complementarity (Table S3).

**Figure 3:**
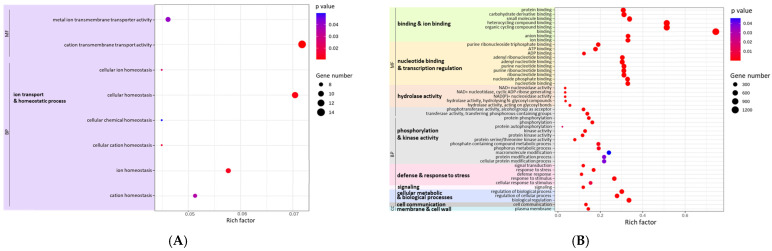
Enriched GO terms associated with DEM-Ts 1 h (**A**) and 24 h (**B**) after PpPep2 treatment of peach leaves. The *x*-axis indicates the rich factor (ratio of genes in the input list that are annotated to the function); and the *y*-axis shows the GO terms grouped into upper families shown in rainbow colors. GO terms are also classified into molecular function (MF), biological process (BP), or cellular component (CC) categories. Dot colors indicate *p*-values, shown in blue (0.05) to red (0.00). Dot radiuses are proportional to gene number (number of genes contributing to each GO term enrichment).

In order to assist the biological interpretation of miRNA changes in response to PpPep2, DEM-Ts were subjected to functional enrichment analysis. Figure 3 depicts the enriched GO terms associated with predicted targets of miRNAs with differential expression one hour and twenty-four hours after peptide treatment.

One hour after PpPep2 treatment, there were just four DEMs. They had up to 322 predicted targets, and they primarily belonged to two molecular functions and six biological process terms that were related to ion transport and homeostasis (Figure 3A). “Cation transmembrane transport activity”, “cellular homeostasis” and, specifically, “ion homeostasis” and “cation homeostasis” were the terms with the highest gene number and rich factor. Effector perception rapidly triggers ionic flux (mainly affecting Ca^2+^, H^+^, K^+^, Cl^−^ and NO^3−^), which leads to extracellular alkalinization that triggers the PTI response [43].

Moreover, we previously described that there was a quick and transient regulation of genes related to “calcium transport” and “calcium signaling” terms in peach leaves treated with PpPep2 in an RNA-Seq transcriptome analysis approach [7]. Our miRNA results suggest that miRNAs (particularly ppeMIR156h, ppe-MIR167a, ppe-MIR535a and/or ppe-MIR535b) play a role in these quick initial steps of PTI.

One day after PpPep2 treatment, there was a stronger miRNA response than at the earlier time, i.e., 39 DEMs. Their corresponding DEM-Ts were associated with 47 GO terms (Figure 3B).

The GO terms with the highest gene number and rich factor were related to binding (specifically, the heterocycling compound, organic compound, carbohydrate, protein and ion binding), and “nucleotide binding and transcription regulation”. Again, there was the regulation of biological processes, mainly linked to the “defense and response to stress” and “phosphorylation and kinase activity”. All these processes are integral to the PTI-like response. Phosphorylation events involving receptors and co-receptors of PAMPs and DAMPs (including PEPR), MAPK cascades and downstream TFs are essential parts of the signal transduction mechanisms. This suggests that miRNAs would have an active role in PTI regulation at these stages. In coherence, there was also the enrichment of GO terms related to signaling, cell communication and membrane and cell wall modifications, also linked to PTI.

### 2.3. miRNAs Participate in PpPep2-Driven Regulation of Genes Involved in the Defense Response

Experimental data on the expression patterns of DEM-Ts in response to 1 μM of PpPep2 treatment were used to identify which of these genes are regulated by PpPep2 at the mRNA level. We previously used RNA-Seq to identify peach leaf differentially expressed genes (DEGs) in response to 1 μM of PpPep2 treatment for 1, 24 and 48 h [7]. The intersection of DEGs with all 2431 DEM-Ts retrieved 176 genes (Table 2), corresponding to 7.2% DEM-Ts and 8.9% PpPep2 DEGs. Therefore, both these genes were regulated by PpPep2 at the mRNA level and are predicted targets of PpPep2-regulated miRNAs. Remarkably, there was at least one DEM-T for every DEM. This supports this subset of DEGs are targets of DEMs; and further suggests that the identified miRNAs are involved in the PpPep2-driven regulation of this specific set of genes.

**Figure 4:**
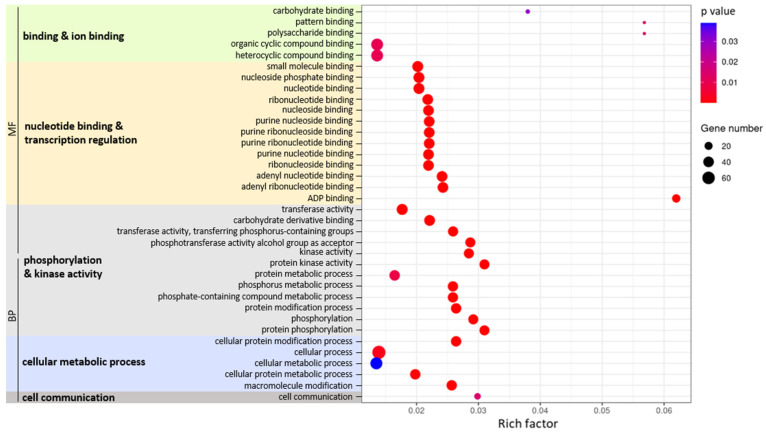
Enriched GO terms associated with DEM-Ts that were differentially expressed after 1 µM of PpPep2 treatment of peach leaves for 1 h, 24 h or 48 h. The *x*-axis indicates the rich factor (ratio of genes in the input list that are annotated to the function), and the *y*-axis shows the GO terms. GO terms are also classified into molecular function (MF) or biological process (BP) categories. Dot color indicate *p*-values, shown in blue (0.05) to red (0.00). Dot radiuses are proportional to the gene number (number of genes contributing to each GO term enrichment).

To gain insight into their role, these 176 DEM-Ts were subjected to enrichment analysis (Figure 4). There were 36 enriched GO terms (*p* < 0.05) that were mainly related to protein phosphorylation and cell communication biological processes and to protein kinase and ADP binding activities. We determined the genes that participated in each enriched GO term, as well as their corresponding DEMs (Table S4).

The enrichment pathways (Figure 4) align with the defense-related roles of PpPep2. We previously used RNA-Seq to show that the topical application of PpPep2 onto peach trees is similar to that of Arabidopsis to its specific AtPeps [17] and mimics the natural PTI response [7]. The initial PTI signal transduction steps involve the phosphorylation of different proteins, notably PEPR and co-receptors and MAP kinases that subsequently regulate the transcription of genes participating in the defense response [3,5,43]. MIR390, MIR482a and MIR8127 were predicted to target a number of genes related to protein phosphorylation and kinase activity. These and MIR482c, e and f were anticipated to target genes with nucleoside or nucleotide binding activity. The topic application of PpPep2 quickly regulates biotic stress receptors, signaling through receptor kinases, and RNA transcription. These results suggest that miRNAs, in particular MIR390, MIR482 and MIR8127, are involved in Pep-driven PTI regulation at these initial stages.

The transcriptome response of peaches to PpPep2 involves genes participating in signaling and hormone metabolism. In agreement, here we find the PpPep2-driven regulation of MIR166e, MIR168, MIR172b and MIR482a, c-3p, e and f, which predictably target genes with cell communication roles.

miRNAs modulate gene expression by binding to specific mRNAs and targeting them for cleavage or by directing translational inhibition at the mRNA level [44]. In accordance, for those inducing mRNA cleavage, we might expect the overexpression of a given miRNA to result in the downregulation of target gene mRNA levels and vice versa. We thus compared the expression patterns of DEMs and those of DEM-Ts that are differentially expressed in peach leaves in response to PpPep2 treatment. Most DEMs had one or more DEM-Ts whose expression patterns in response to PpPep2 were diametrically opposed to that of the DEM (Table 2). Although there is no report on the role of these DEMs nor experimental proof of their target genes in peach trees, some have been studied in other species. MIR genes, such as MIR156 [45,46,47] and MIR390 [45,48,49,50], are involved in plant development and the response to stress and participate in the growth and defense trade-off. Others, e.g., MIR482 and MIR395, seem to be more specifically involved in the plant defense responses [51,52].

We found that ppe-MIR156g was induced up to six and sevenfold in peach leaves treated with PpPep2 for 1 h and 24 h, respectively, being the most strongly regulated miRNA in these conditions. One of its DEM-Ts is the Squamosa Promoter-Binding-Like (SPL) protein Teosinte Glume Architecture 1 (TGA1) (Table 2), whose mRNA levels were also regulated by PpPep2 in the same peach system [7]. In agreement, ZmMIR156g proved to target TGA1 in maize [47,53]. The evolutionary conserved MIR156 family members are known to target SPLs, whose mRNA levels increase during shoot development as MIR156 expression gradually declines in abundance [45]. MIR156 family members are induced to arrest development under stress conditions, and their miRNAs are suppressed when the plants are returned to favorable conditions, allowing the developmental transition to be accelerated [46].

In peach leaves, we found a negative relation between ppe-MIR390 and its DEM-T, Leucine-Rich Repeat, upon the application of PpPep2 for 1 h and 24 h. A similar relation was described in apple tree, where mdm-miR390a overexpression enhanced the sensitivity to Alternaria infection through the downregulation of MdLRR8 and the LRR-LRK serine/threonine-protein kinase MdRPK2. This way, the fungal induction of apple mdm-miR390a increases the susceptibility of the plant host to infection [48]. Our results suggest that endogenous peptides such as PpPep2 may contribute to pathogen resistance by the downregulation of ppe-MIR390 and the parallel upregulation of LRR. The downregulation of MIR390 in response to stress results in the suppression of growth and yield reduction in rice and *Arabidopsis* [49,54], which might as well be an effect of PpPep2.

The MIR482 family is highly represented among PpPep2-regulated MIRs, with four DEMs out of six annotated miRNAs (MIR482a-3p, MIR482c-3p, MIR482e, MIR482f), representing 66% of the family. Various PpPep2-regulated Resistance (R) genes were among MIR482 DEM-Ts, e.g., putative disease resistance protein RGA3 (ppe-MIR482e target), disease resistance RPP13-like protein 1 (ppe-MIR482f target) and the receptor-like protein kinase FERONIA (ppe-MIR482a-3p target). As expected for miRNA regulation, the expression patterns of these miRNAs and their predicted targets are inversely related (Table 2). Thus, peach MIR482 seems to be related to regulation of the defense response. In agreement, previous studies showed that MIR482 genes target members of different R gene families in apple, cotton, soybean, *Medicago truncatula* and various Solanaceae species, e.g., tomato and potato [48,55,56,57]. In *Arabidopsis*, MIR482 regulates an NBS-LRR-type R protein that inhibits jasmonic acid (JA) signaling and positively regulates immunity [51].

Up to 27% of DEMs identified in this study were classified in the MIR395 family; nine out of fifteen annotated peach MIR395 members were DEMs (i.e., ppe-MIR395a, -c, -f, -i, -j, -k, -l, -m and -n). All nine ppe-MIR395 family members were downregulated after the PpPep2 application and shared a common mature sequence, which was predicted to target Sulfate Transporter 2.1 (SULTR2) and ATP Sulfurylase (ATPS) transcripts (Table 2). These sulfate-related genes were upregulated one day after PpPep2 treatment. SULTR2 and ATPS are involved in sulfate assimilation into glutathione, providing antioxidant protection against oxygen free radical-mediated damage brought on by pathogens [52,58]. This way, ppe-MIR395 seems to display a role in peach disease resistance, as previously observed in A. thaliana and eggplant [52,59]. In contrast to the Arabidopsis response to the PAMP flg22, MIR395 is among the most strongly regulated miRNAs in response to the exogenous application of the DAMP PpPep2 onto peach.

Taken together, our results enhance the understanding of PpPep2 as a phytosanitary tool to boost peach tree defenses against pathogens, consolidating it as a promising plant disease management tool. PpPep2-driven miRNA regulation is compatible with pathogen defense stimulation and also the developmental arrest associated with stress resistance, which again mimics plant natural defense systems.

## 3. Materials and Methods

### 3.1. Plant Material and Peptide Treatments

Peach juvenile plants (*Prunus persica* var. Big Top) were produced using in vitro technology and grown in individual small pots by a professional grower (Agromillora Iberia S.A., Barcelona, Spain). Plant treatments were carried out as previously described [7]. Briefly, plants were acclimatized for two weeks in a glasshouse (21 °C, a 16/8 h light/dark photoperiod and 60% RH) prior to treatments with 1 µM of PpPep2 (YVQRITLRAARPEISTGSGAQTN, the full-length mature peptide of MW600837 [10,60], chemically synthesized by Caslo ApS (Lyngby, Denmark) with purity above 95% and the identity confirmed by MALDI-TOF. The five youngest fully expanded leaves of each plant were labeled, and PpPep2 was sprayed onto both abaxial and adaxial leaf surfaces. Plants were incubated under standard conditions in the glasshouse for 0, 1 and 24 h. Leaves were detached, and the leaf blades were immediately frozen in liquid nitrogen. The experimental design consisted of three replicates of nine plants per treatment.

### 3.2. RNA Extraction and Illumina Sequencing

RNA was extracted from a 200 mg aliquot of a ground leaf sample through a TRIzol-based procedure (Invitrogen Life Technologies, Carlsbad, CA, USA) followed by DNAse I (Ambion, Grand Island, NY, USA) digestion and purification with RNeasy MinElute Cleanup Kit (Qiagen, Sollentuna, Sweden). The estimation of RNA concentration was conducted using a NanoDrop ND1000 spectrophotometer (Nanodrop Technologies, Wilmington, DE, USA).

miRNA-Seq was carried out at the Centre Nacional d’Anàlisi Genòmica (CNAG) (Barcelona, Spain). Libraries were prepared using the NEBNext^®^ Small RNA Library Prep Set for Illumina^®^ kit (ref. E7330) (New England Biolabs, Ipswich, MA, USA) according to the manufacturer’s protocol. Briefly, RNA was subjected to adaptor 3′and 5′ ligation and first strand cDNA synthesis. Then, DNA fragments with adapter molecules on both ends were selectively enriched by a PCR using NEBNext^®^ Multiplex Oligos for Illumina (Index Primers Set 1, ref. E7335, Index Primers Set 2, ref. E7500, Index Primers Set 3, ref. E7710 and Index Primers Set 4, ref. E7730) (New England Biolabs, Ipswich, MA, USA). Purification steps were then performed using AgenCourt AMPure XP beads (ref. A63882) (Beckman Coulter, Brea, CA, USA), and final libraries were analyzed using the Agilent Bioanalyzer (ref. 5067-4626) (Agilent Technologies, Santa Clara, CA, USA) to estimate the quantity and check size distribution. A pool was conducted to perform size selection using 6% Novex TBE PAGE Gels (ref. EC6265BOX) (ThermoFisher Scientifics, Waltham, MA, USA), and, then, the final pool was quantified by qPCR using the KAPA Library Quantification Kit (ref. KK4835, KapaBiosystems) (Roche, Basel, Switzerland) prior to amplification with Illumina’s cBot. Libraries were sequenced 1 × 50 + 8 bp on the Illumina HiSeq2500 device (Illumina, San Diego, CA USA).

### 3.3. Bioinformatics Analysis

Small RNA-Seq reads were trimmed using Trim Galore version 0.6.6 [61] to remove adapter sequences and low-quality bases. Trimmed reads were mapped against *Prunus persica* reference genome (Prunus_persica_NCBIv2.38) using STAR software version 2.5.3a [62] with ENCODE parameters for small reads. Annotated genes were quantified using release 48 of Ensembl Plants annotation with RSEM version 1.3.0 [63], modifying the default parameter seed length parameter to 16 to improve alignment for small RNA-seq.

Differential expression analysis of annotated pre_miRNA gene biotypes between time points was conducted using the DESeq2 v1.26.0 R package [38]. DESeq2 performs independent filtering by default, using the mean of normalized counts as a filter statistic. Significance testing was performed with the Wald test, and *p*-values were adjusted using the Benjamini–Hochberg method for multiple test correction, and genes were considered statistically significant with an adjusted *p*-value below 0.05.

The miRNA gene target prediction was carried out using psRNATarget [40], which evaluates the complementarity between miRNA and target mRNA sequences and the accessibility of the mRNA based on its secondary structure with a scoring schema. The reliability of psRNATarget predictions is supported by the high recall rates reported in its benchmark analyses. According to the Nucleic Acids Research publication of the tool, psRNATarget achieved a recall rate of 97.3% for Arabidopsis thaliana and 82.7% for rice, demonstrating its capacity to accurately predict miRNA-target interactions across diverse plant species.

Gene Ontology enrichment analysis of miRNA-predicted gene targets was performed using the gprofiler2 v0.1.8 R package [64]. The analysis relies on a cumulative hypergeometric probability (Fisher’s one-tail test) to find the over-representation of GO terms from the Ensembl database within the significant gene target list. Principal component analysis (PCA) was carried out on normalized counts using the prcomp R function to assess sample variability. Visualization, including PCA, volcano plots and enrichment dot plots were generated using the R package ggplot2 v3.5.1 [65].

## 4. Conclusions

Here, we further analyze the mode of action of PpPep2, which causes PTI-like transcriptome reprogramming and enhances resistance to *Xap* in *P. persica* [7,10]. One hour and one day after peptide application, there were expression changes in up to 7% of peach genes. Furthermore, 1 day of pretreatment with chemically synthesized PpPep2 before exposure to massive doses of *Xap* results in about 50% reduction in the disease symptoms.

The miRNA-Seq analysis of the peach tree response to the application of PpPep2 showed that miRNAs participate in the Pep-triggered defense response of plants. About 15% of miRNA genes were regulated by PpPep2 within 24 h. For every DEM, there were some DEM-Ts that were regulated in response to the same PpPep2 treatment. Inversely, about 10% of the PpPep2-regulated protein-coding genes were DEM-Ts. These miRNAs appear directly involved in the PpPep2-driven regulation of their corresponding target genes, which are related to PTI-like processes. A number of experiments carried out in other plant species support the regulatory role of several of these miRNAs towards their predicted target genes in the context of defense and their balance versus growth.

Additionally, our findings indicate that the topical application of Peps activates plant immune systems, involving not only transcriptomic changes but also miRNA regulatory pathways. The parallelism between the plant response to the Pep topic application and natural defense mechanisms indicates its mechanism of action and the lack of safety concerns related to this natural phytosanitary product.

## Figures and Tables

**Table 1:** *P. persica* leaf DEMs 1 and 24 h after PpPep2 topic application. For every DEM, the Ensembl (ID) and miRBase (miRNA) IDs are shown, together with their predicted mature sequences, average normalized counts in control (t0) and PpPep2-treated samples for one (t1) and 24 (t24) h and expression changes at every time point compared to t0 (log2 fold-values, t1_t0 and t24_t0).

ID	miRNA		Predicted Mature Sequence			Normalized Counts				t1_t0	t24_t0
			Strand	Sequence 5′-3′		t0	t1	t24			
ENSRNA049996926	ppe-miR156g		5p	UUGACAGAAGAUAGAGAGCAC		1	93	170		6.0	6.8
ENSRNA049996633	ppe-miR171c		5p	UGAUUGAGCCGUGCCAAUAUC		4	16	16		2.1	2.1
ENSRNA049995981	ppe-miR7122b		3p	CCGUGUUUCCUUGUAUAAAG		115	301	368		1.4	1.7
			5p	UUAUACAAUGAAAUCACGGUCG							
ENSRNA049995989	ppe-miR7122a		3p	GCCGUGUUUCUUUGUAUAAAG		2506	5006	6695		1.0	1.4
			5p	UUAUACAAUGAAAUCACGGCCG							
ENSRNA049996187	ppe-miR482a		3p	UUUCCGAAACCUCCCAUUCCAA		324	501	620		0.6	0.9
			5p	GGGUGAGAGGUUGCCGGAAAGA							
ENSRNA050003821	ppe-miR396b		5p	UUCCACAGCUUUCUUGAACUU		8203	12,003	14,798		0.5	0.9
ENSRNA049996209	ppe-miR482f		3p	UCUUUCCUACUCCACCCAUUCC		3215	4544	6556		0.5	1.0
ENSRNA049996042	ppe-miR403		3p	UUAGAUUCACGCACAAACUCG		1307	1823	2510		0.5	0.9
ENSRNA049996194	ppe-miR482e		3p	UUGCCUAUUCCUCCCAUGCCAA		534	608	825		0.2	0.6
ENSRNA049996910	ppe-miR162		3p	UCGAUAAACCUCUGCAUCCAG		2901	3101	4694		0.1	0.7
ENSRNA049996592	ppe-miR171a		3p	UGAUUGAGCCGUGCCAAUAUC		74	99	111		0.4	0.6
ENSRNA050003799	ppe-miR159		3p	UUUGGAUUGAAGGGAGCUCUA		82	106	128		0.4	0.6
ENSRNA049997214	ppe-miR167a		5p	UGAAGCUGCCAGCAUGAUCUA		200	495	284		1.3	0.5
ENSRNA049996319	ppe-miR8127		3p	UUCAAAGGGUACAUCCACAGU		1745	1803	1372		0.0	−0.3
			5p	CAACUGUGGACAUACCCUUUG							
ENSRNA049996833	ppe-miR168		5p	UCGCUUGGUGCAGGUCGGGAA		788	648	485		−0.3	−0.7
ENSRNA049997189	ppe-miR535b		5p	UGACGACGAGAGAGAGCACGC		466	178	199		−1.4	−1.2
ENSRNA049996546	ppe-miR482c		3p	UUCCCAAGCCCGCCCAUUCCAA		4406	2720	1915		−0.7	−1.2
			5p	UUCCCAAGCCCGCCCAUUCCAA							
ENSRNA049996130	ppe-miR395c		3p	CUGAAGUGUUUGGGGGAACUC		108	62	49		−0.8	−1.1
ENSRNA049996345	ppe-miR172b		3p	AGAAUCUUGAUGAUGCUGCAU		113	116	43		0.0	−1.4
ENSRNA050003783	ppe-miR390		5p	AAGCUCAGGAGGGAUAGCGCC		416	299	152		−0.5	−1.4
ENSRNA049996145	ppe-MIR395n		3p	CUGAAGUGUUUGGGGGAACUC		233	185	84		−0.3	−1.5
ENSRNA049996894	ppe-miR166e		3p	UCGGACCAGGCUUCAUUCCCC		2574	1091	801		−1.2	−1.7
ENSRNA049996107	ppe-miR395a		3p	CUGAAGUGUUUGGGGGGACCC		6318	3680	1792		−0.8	−1.8
			5p	GUUCCCUCAAACACUUCAUU							
ENSRNA049997194	ppe-miR535a		5p	UGACAACGAGAGAGAGCACGC		167	37	43		−2.2	−1.9
ENSRNA049996563	ppe-miR156h		5p	UUGACAGAAGAUAGAGAGCAC		693	289	188		−1.3	−1.9
ENSRNA049996028	ppe-miR169d		5p	UGAGCCAAGGAUGACUUGCCA		48	27	10		−0.8	−2.2
ENSRNA049996100	ppe-miR395l		3p	CUGAAGUGUUUGGGGGAACUC		1193	558	252		−1.1	−2.2
ENSRNA050003167	ppe-miR530		5p	UCUGCAUUUGCACCUGCACCU		391	178	76		−1.1	−2.4
ENSRNA049996115	ppe-miR395j		3p	CUGAAGUGUUUGGGGGAACUC		40	21	5		−0.9	−3.0
ENSRNA049996137	ppe-miR395i		3p	CUGAAGUGUUUGGGGGAACUC		50	19	6		−1.4	−3.1
ENSRNA049996172	ppe-miR395k		3p	CUGAAGUGUUUGGGGGAACUC		376	233	36		−0.7	−3.4
ENSRNA049996180	ppe-miR395f		3p	CUGAAGUGUUUGGGGGAACUC		53	20	4		−1.4	−3.8
ENSRNA049996069	ppe-miR395m		3p	CUGAAGUGUUUGGGGGAACUC		17	7	1		−1.3	−3.9

Color codes correspond to statistically up—(red) and down—(blue) regulated miRNAs in response to PpPep2, DESeq2 v1.26.0 adj. *p* < 0.05. Color intensity indicates fold change. Numbers in gray font indicates not statistically significant. Note that the last letter in every miRNA code differentiates members within the same miRNA family. Table S2 shows normalized counts and statistics for all samples and replicates.

**Table 2:** Expression patterns of DEMs and predicted target genes that are differentially expressed in peach leaves in response to PpPep2 treatment. Only those that have opposing expression patterns and up to two predicted target genes per DEM (i.e., those with the lowest *p*-value in target gene prediction) are displayed. t1_t0, t24_t0, and t48_t0 indicate gene expression log2fold-change values at 1, 24, and 48 h after PpPep2 treatment compared to untreated samples, respectively.

DEM					Predicted Target Gene 1						Predicted Target Gene 2				
miRNA	t1_t0	t24_t0	# DEG Targets		Target ID	Target Description	t1_t0	t24_t0	t48_t0		Target ID	Target Description	t1_t0	t24_t0	t48_t0
ppe-MIR156g	6.0	6.8	2		PRUPE.5G208300	Teosinte glume architecture 1	0.7	−0.3	0.3						
ppe-MIR171c	2.1	2.1	4		PRUPE.2G274300	Hexose carrier protein HEX6	1.5	0.3	−0.6						
ppe-miR7122b-3p	1.4	1.7	3												
ppe-miR7122b-5p	1.4	1.7	6		PRUPE.3G227700	Uncharacterized LOC18783989	−0.5	1.1	0.5						
ppe-miR7122a-3p	1.0	1.4	5		PRUPE.6G281500	Receptor-like protein 12	2.0	0.0	0.0		PRUPE.3G039200	Linoleate 13S-lipoxygenase 3-1, chloroplastic	1.9	0.5	0.2
ppe-miR7122a-5p	1.0	1.4	4		PRUPE.1G317500	Lipid phosphate phosphatase 2	0.9	−0.1	−0.2						
ppe-MIR482f	0.5	1.0	11		PRUPE.2G022400	Putative disease resistance RPP13-like protein 1	1.0	−0.6	−0.3						
ppe-miR403	0.5	0.9	2		PRUPE.1G525900	Pentatricopeptide repeat-containing protein At5g27460	1.5	0.1	−0.1		PRUPE.1G385000	Hypothetical protein PRUPE.1G385000	1.7	−0.2	−0.7
ppe-miR482a-3p	0.6	0.9	15		PRUPE.6G295900	Receptor-like protein kinase FERONIA	1.1	−0.5	0.4						
ppe-miR482a-5p	0.6	0.9	10												
ppe-miR396b	0.5	0.9	9		PRUPE.5G013100	Abscisic acid 8′-hydroxylase 1	3.1	−0.7	1.1						
ppe-miR162	0.1	0.7	3		PRUPE.1G385500	Hypothetical protein PRUPE.1G385500	1.0	−0.1	−0.8						
ppe-miR159	0.4	0.6	5		PRUPE.6G073300	Scarecrow-like protein 30	3.4	0.3	−0.8						
ppe-miR482e	0.2	0.6	10		PRUPE.4G284000	Putative disease resistance protein RGA3	0.8	−0.5	−0.4						
ppe-miR171a	0.4	0.6	4		PRUPE.2G274300	Hexose carrier protein HEX6	1.5	0.3	−0.6						
ppe-MIR167a	1.3	0.5	9		PRUPE.2G290200	Protein DETOXIFICATION 49	4.7	0.8	−0.4						
ppe-miR8127-3p	0.0	−0.3	2												
ppe-miR8127-5p	0.0	−0.3	4												
ppe-miR168	−0.3	−0.7	3		PRUPE.7G053500	Glycerol-3-phosphate dehydrogenase [NAD(^+^)] GPDHC1, cytosolic	−0.3	0.8	0.6						
ppe-MIR395c	−0.8	−1.1	15		PRUPE.6G180002	Sulfate transporter 2.1	0.2	−0.9	0.5		PRUPE.1G023002	ATP sulfurylase 1, chloroplastic	0.2	−0.5	0.9
ppe-MIR482c-3p	−0.7	−1.2	12												
ppe-miR482c-5p	−0.7	−1.2	15		PRUPE.7G243500	SPX domain-containing protein 1	0.7	2.2	1.0						
ppe-MIR535b	−1.4	−1.2	10		PRUPE.4G158000	Nematode-induced LRR-RLK 2 (NILR1)	2.1	−0.7	−0.8						
ppe-MIR172b	0.0	−1.4	12												
ppe-MIR390	−0.5	−1.4	10		PRUPE.4G121900	Receptor-like protein 12	4.2	0.3	0.0						
ppe-MIR395n	−0.3	−1.5	15		PRUPE.6G180009	Sulfate transporter 2.1	0.2	−0.9	0.5		PRUPE.1G023009	ATP sulfurylase 1, chloroplastic	0.2	−0.5	0.9
ppe-MIR166e	−1.2	−1.7	5		PRUPE.7G041600	Two-component response regulator ORR9	−0.9	0.1	0.6						
ppe-MIR395a-3p	−0.8	−1.8	7		PRUPE.6G180000	Sulfate transporter 2.1	0.2	−0.9	0.5		PRUPE.1G023000	ATP sulfurylase 1, chloroplastic	0.2	−0.5	0.9
ppe-miR395a-5p	−0.8	−1.8	4		PRUPE.6G180001	Sulfate transporter 2.1	0.2	−0.9	0.5		PRUPE.1G023001	ATP sulfurylase 1, chloroplastic	0.2	−0.5	0.9
ppe-MIR535a	−2.2	−1.9	8		PRUPE.8G263900	Probable pectinesterase/pectinesterase inhibitor 7	4.7	1.4	1.4						
ppe-MIR156h	−1.3	−1.9	2		PRUPE.4G089000	Cationic amino acid transporter 1	1.6	0.7	0.7						
ppe-MIR395l	−1.1	−2.2	15		PRUPE.6G180007	Sulfate transporter 2.1	0.2	−0.9	0.5		PRUPE.1G023007	ATP sulfurylase 1, chloroplastic	0.2	−0.5	0.9
ppe-MIR169d	−0.8	−2.2	7		PRUPE.7G093100	Nuclear transcription factor Y subunit A-10 (NFYA10)	0.1	0.1	0.3						
ppe-miR530	−1.1	−2.4	1												
ppe-MIR395j	−0.9	−3.0	15		PRUPE.6G180005	Sulfate transporter 2.1	0.2	−0.9	0.5		PRUPE.1G023005	ATP sulfurylase 1, chloroplastic	0.2	−0.5	0.9
ppe-MIR395i	−1.4	−3.1	15		PRUPE.6G180004	Sulfate transporter 2.1	0.2	−0.9	0.5		PRUPE.1G023004	ATP sulfurylase 1, chloroplastic	0.2	−0.5	0.9
ppe-MIR395k	−0.7	−3.4	15		PRUPE.6G180006	Sulfate transporter 2.1	0.2	−0.9	0.5		PRUPE.1G023006	ATP sulfurylase 1, chloroplastic	0.2	−0.5	0.9
ppe-MIR395f	−1.4	−3.8	15		PRUPE.6G180003	Sulfate transporter 2.1	0.2	−0.9	0.5		PRUPE.1G023003	ATP sulfurylase 1, chloroplastic	0.2	−0.5	0.9
ppe-MIR395m	−1.3	−3.9	15		PRUPE.6G180008	Sulfate transporter 2.1	0.2	−0.9	0.5		PRUPE.1G023008	ATP sulfurylase 1, chloroplastic	0.2	−0.5	0.9

Color codes indicate statistically up- (red) and down- (blue) regulated miRNAs and predicted target genes in response to PpPep2. # DEG targets, number of predicted miRNA targets that are differentially expressed upon PpPep2 treatment.

## Data Availability

Data supporting transcriptome analysis are available in the Gene Expression Omnibus (GEO) repository, record GSE214135, enter token slshgkouhfajdyp into the box (https://www.ncbi.nlm.nih.gov/geo/query/acc.cgi?acc=GSE214135; accessed on 1 December 2024).

## Supplementary Materials

The following supporting information can be downloaded at https://www.mdpi.com/article/10.3390/ijms252313099/s1.
