# Supplementary material for: The PpPep2-Triggered PTI-like Response in Peach Trees Is Mediated by miRNAs
Source: Int J Mol Sci. 2024 Dec 5;25(23):13099. doi: 10.3390/ijms252313099 (PMC11642718; doi:10.3390/ijms252313099)
Supplement: Supplementary file 1 [file ijms-25-13099-s001.zip › Foix et al_Supplementary material table of contents_revision.pdf]

Supplementary Materials

The PpPep2-triggered PTI-like response in peach trees is mediated by miRNAs

Laura Foix [1,2], Maria Pla [2], Anna Esteve-Codina [3,4], Beatriz Martín-Mur [3], and Anna Nadal [2,*]

[1] BETA Tech. Center (TECNIO Network), University of Vic-Central University of Catalonia (UVic-UCC), Carretera de Roda 70, 08500 Vic, Spain
[2] Institute for Agricultural and Food Technology, University of Girona, Campus Montilivi (EPS-1), 17003 Girona, Spain
[3] CNAG-CRG, Centre for Genomic Regulation, Barcelona Institute of Science and Technology, 08028 Barcelona, Spain.
[4] Universitat Pompeu Fabra (UPF), Barcelona, Spain.

* Correspondence: anna.nadal@udg.edu

Table of contents

Table S1. Statistics summary of miRNA-Seq.

Quality control and mapping statistics of miRNA analysis. Obtaining 117, 267, 647 raw reads that corresponded to an average of 12, 11 and 16 million reads 1 and 24 h after PpPep2 application and control, respectively.

Excel file.

Table S2. Normalized counts and statistics of miRNA-Seq.

Normalized counts and statistics of miRNA analysis of *P. persica* leaf samples treated with Ppep2 for 1 and 24 hours. Values corresponding to all samples and replicates are depicted.

Excel file.

Table S3. *P. persica* leaf DEMs (differentially expressed miRNAs) 1h and 24h after PpPep2 topic application.

A range of 45 to 117 deduced targets were predicted for each DEM, with varying degrees of targeting probability based on miRNA and target complementarity. For every DEM, the number of potential targets and their Ensembl (ID) codes are shown.

Excel file.

Table S4. Enriched GO terms associated to DEM predicted target genes differentially expressed after 1 μM PpPep2 treatment of peach leaves for 1h, 24h or 48 h (DEG).

GO terms are classified into molecular function (MF), biological process (BP) or cellular component (CC) categories. For each enriched GO term, the description, the adjusted *p-value*, and the number of genes in the DEG input list and in the background reference list are shown. Also shown are the specific DEMs targeting genes that contribute to each GO term (black squares).

Excel file.
